# The Relationship Between Accuracy of Numerical Magnitude Comparisons and Children’s Arithmetic Ability: A Study in Iranian Primary School Children

**DOI:** 10.5964/ejop.v12i4.1175

**Published:** 2016-11-18

**Authors:** Hamdollah Manzari Tavakoli

**Affiliations:** a Department of Education, Kerman Branch, Islamic Azad University, Kerman, Iran; University of South Wales, Pontypridd, United Kingdom

**Keywords:** symbolic and non-symbolic numerical magnitude comparison, arithmetic ability, working memory, processing speed, long-term memory

## Abstract

The relationship between children’s accuracy during numerical magnitude comparisons and arithmetic ability has been investigated by many researchers. Contradictory results have been reported from these studies due to the use of many different tasks and indices to determine the accuracy of numerical magnitude comparisons. In the light of this inconsistency among measurement techniques, the present study aimed to investigate this relationship among Iranian second grade children (n = 113) using a pre-established test (known as the Numeracy Screener) to measure numerical magnitude comparison accuracy. The results revealed that both the symbolic and non-symbolic items of the Numeracy Screener significantly correlated with arithmetic ability. However, after controlling for the effect of working memory, processing speed, and long-term memory, only performance on symbolic items accounted for the unique variances in children’s arithmetic ability. Furthermore, while working memory uniquely contributed to arithmetic ability in one-and two-digit arithmetic problem solving, processing speed uniquely explained only the variance in single-digit arithmetic skills and long-term memory did not contribute to any significant additional variance for one-digit or two-digit arithmetic problem solving.

## Introduction

Attaining arithmetic ability is a main academic skill children learn during elementary school years. It provides the foundation for learning higher forms of mathematics such as fraction, ratio, proportion, percentages and algebra. Successes in daily life, society, and labor increasingly depend on this quantitative skill. Difficulties in performing arithmetic skills may face children with substantial problems in educational setting and negatively interfere with their daily experiences throughout life. In order to provide scientifically appropriate remedial teaching strategies for children suffering from mathematical difficulties, a number of researchers tried to find both specific numerical and general cognitive factors underlying arithmetic ability (e.g., Caviola, Mammarella, Lucangeli, & Cornoldi, 2014; Fuchs et al., 2010; Krajewski & Schneider, 2009; Östergren & Träff, 2013; Passolunghi & Lanfranchi, 2012; Träff, 2013; Vanbinst, Ansari, Ghesquière, & De Smedt, 2016). However, contradictory results have been reported from these studies due to the use of many different tasks and indices to determine the accuracy of numerical magnitude comparisons and general cognitive factors.

We aimed to measure performance on symbolic and non-symbolic numerical magnitude comparisons using the Numeracy Screener, working memory, processing speed, and long-term memory and determine their contribution to arithmetic ability among second-grade Iranian children. The main research question is whether variability in children’s scores on symbolic or non-symbolic items of the Numeracy Screener could uniquely explain the variability in single-digit or two-digit arithmetic problem solving after controlling for the effect of working memory, processing speed, and long-term memory, as these are domain-general cognitive skills related to mathematical abilities. Before detailing the specifics of the method used, the literature describing numerical magnitude comparison and its contribution to children’s arithmetic ability will be reviewed. In addition, the roles of working memory, processing speed, and long-term memory as three domain-general cognitive abilities influencing children’s arithmetic development will be briefly explained.

### Non-Symbolic and Symbolic Numerical Magnitude Comparison and Their Relationship to Arithmetic

In recent years, psychologists have proposed that humans and many other species have an inherent sense of quantity known as “Approximate Number Sense” (ANS). This mental number representation system allows approximate estimation of quantities without counting. When observing different non-symbolic magnitudes (e.g., arrays of dots), our ANS enables us to implicitly discriminate between smaller and larger ones (e.g., choosing the more numerous dot array). Our accuracy performing these non-symbolic comparisons dramatically changes from infancy to adulthood. (Barth, Kanwisher, & Spelke, 2003; Halberda & Feigenson, 2008; Lipton & Spelke, 2003; Xu & Spelke, 2000). This improvement in discrimination accuracy suggests that infants and young children are much more imprecise in their ability to approximate quantity via non-symbolic representations than are older children and adults.

After going to school, children acquire a new symbolic system for representation of magnitudes. Similar to the pre-existing non-symbolic system, the new system allows children to manipulate or compare magnitudes, but in symbolic forms such as words that represent numbers or Arabic digits. To compare quantities using symbolic representations (e.g., Arabic digit comparison), researchers assumed that children need to map the acquired symbolic system onto an already-existing non-symbolic system (Deheane, 2011; Nosworthy, Bugden, Archibald, Evans, & Ansari, 2013; Price et al., 2012). In other words, processing of non-symbolic magnitudes is the precursor to the processing and comparison of symbolic representations of magnitude.

During recent decades, it has been assumed that numerical magnitude processing is related to arithmetic development in children. De Smedt, Verschaffel, and Ghesquière (2009) provided theoretical arguments and behavioral evidence that sufficiently support this assumption. First, understanding numerical magnitudes might help children use counting procedures to solve arithmetic problems. When solving arithmetic problems involving single digits (e.g., 7+3), children initially count both addends to find the solution (1, 2, 3, …, 7, 8, 9, 10). Gradually, they learn to use a more advanced strategy, known as counting on or min procedure, in which children start counting from the larger addend to solve the problem (e.g., counting 7, 8, 9, 10 to solve 7+3). A child’s ability to make a decision on the larger addend is dependent on his or her understanding of numerical magnitudes (De Smedt et al., 2009). Neuroimaging studies have also revealed that numerical magnitude processing is important for mathematical achievement. Researchers have found that the intraparietal sulcus, an area of the brain that is dedicated to the processing of numerical magnitude comparisons (Holloway & Ansari, 2010; Lyons, Ansari, & Beilock, 2015) is consistently activated during arithmetic problem solving (e.g., Barnea-Goraly, Eliez, Menon, Bammer, & Reiss, 2005; Bugden, Price, McLean, & Ansari, 2012; Isaacs, Edmonds, Lucas, & Gadian, 2001; Price, Mazzocco, & Ansari, 2013). Moreover, structural and functional abnormalities in the intraparietal sulcus have been found in children with mathematical learning disabilities (Barnea-Goraly et al., 2005; De Smedt, Holloway, & Ansari, 2011; Heine et al., 2013; Price, Holloway, Räsänen, Vesterinen, & Ansari, 2007). Third, a number of researchers studying numerical magnitude processing in children with a mathematical learning disability or with developmental dyscalculia showed that these children have specific deficits related to the understanding and processing of numerical magnitudes (e.g., Brankaer, Ghesquière, & De Smedt, 2014; Defever, De Smedt, & Reynvoet, 2013; Geary, Hoard, Nugent, & Byrd-Craven, 2008; Landerl, Bevan, & Butterworth, 2004; Rousselle & Noël, 2007).

In building a body of behavioral and theoretical evidence related to numerical magnitude processing in early life and its correlation with arithmetic ability in children, several researchers have used different numeracy comparison computerized tasks to explore the relationship between both symbolic and non-symbolic numerical magnitude comparison and previous, concurrent, or future arithmetic ability in children. However, these researchers reported contrasting results (see Chen & Li, 2014; De Smedt et al., 2013 for a review). While a number of researchers have found significant correlations between non-symbolic numerical comparison (dot arrays) and arithmetic skills (e.g., Bonny & Lourenco, 2013; Halberda, Mazzocco, & Feigenson, 2008; Inglis, Attridge, Batchelor, & Gilmore, 2011; Linsen, Verschaffel, Reynvoet, & De Smedt, 2014; Mazzocco, Feigenson, & Halberda, 2011; Mundy & Gilmore, 2009; Vanbinst, Ghesquière, & De Smedt, 2015), other have not (e.g., Holloway & Ansari, 2009; Kolkman, Kroesbergen, & Leseman, 2013; Lonnemann, Linkersdörfer, Hasselhorn, & Lindberg, 2011; Mundy & Gilmore, 2009; Nosworthy et al., 2013; Sasanguie, Göbel, Moll, Smets, & Reynvoet, 2013; Sasanguie, Van den Bussche, & Reynvoet, 2012; Soltész, Szűcs, & Szűcs, 2010; Vanbinst, Ghesquière, & De Smedt, 2012). Furthermore, a large number of studies have reported significant correlations between symbolic numerical comparison (Arabic digits) and arithmetic abilities (e.g., Holloway & Ansari, 2009; Kolkman et al., 2013; Lonnemann et al., 2011; Mundy & Gilmore, 2009; Nosworthy et al., 2013; Sasanguie et al., 2012; Vanbinst et al., 2015); however, a smaller number of studies did not find these significant relationships (e.g., Sasanguie et al., 2012).

A possible explanation of the contrasting results reporting the relationship between numerical knowledge and arithmetic ability is that no commonly accepted, identical version of the magnitude comparison task exists (De Smedt et al., 2013). To assess individuals’ numerical comparison performance, many elements of the tasks have varied, including the sizes of the dot arrays, the ways of controlling the visual characteristics of the dots, the length of time that the subjects could use while determining the value difference between the two displays, and (most importantly) the variety of indices used to measure performance acuity (De Smedt, Noël, Gilmore, & Ansari, 2013; Inglis & Gilmore, 2014). The four common indices usually used to measure comparison performance acuity are accuracy, the Weber fraction, or the effects of either numerical distance or ratio on the accuracy or reaction time of subjects. Using the accuracy index, researchers simply report the proportion of trial participants who answered correctly in a given period of time (Nosworthy et al., 2013) or without any time limitation (e.g., Fuhs & McNeil, 2013; Gilmore, Attridge, & Inglis, 2011; Kolkman et al., 2013; Lourenco, Bonny, Fernandez, & Rao, 2012). The Weber fraction index (w) measures the precision of an individual’s ANS representation; the representation becomes more precise as w approaches zero (Inglis & Gilmore, 2014). Finally, when numerical distance or ratio effect are used to measure the acuity of comparison, the slopes of lines or curves that relate accuracy or reaction time to ratio or numerical distance are calculated (Inglis & Gilmore, 2014). Assuming that these four indices measure the same underlying property, some researchers have just used the accuracy index (Fuhs & McNeil, 2013; Gilmore et al., 2011; Kolkman et al., 2013; Lourenco et al., 2012; Nosworthy et al., 2013), while others have relied upon the Weber fraction, the numerical distance or ratio effect (Bugden et al., 2012; Holloway & Ansari, 2009; Lourenco et al., 2012; Maloney, Risko, Preston, Ansari, & Fugelsang, 2010; Merkley & Ansari, 2010; Price et al., 2012). However, it has been shown that these are not interchangeable constructs and reliable tools must assess the same construct for cross-comparison (Inglis & Gilmore, 2014; Lindskog, Winman, Juslin, & Poom, 2013). Inglis and Gilmore (2014) investigated the psychometric properties of (and interrelation between) these different indices. They found that numerical distance or ratio effect has poor reliability with retesting in the same group and has no relationship with either Weber fractions or accuracy. Moreover, the researchers showed that Weber fractions have lower retest reliability than accuracy (Inglis & Gilmore, 2014). In response to these findings, the researchers recommended indexing acuity on an individual’s numerical comparison by reporting just their accuracy figures (Inglis & Gilmore, 2014).

An identical test intended to use just the accuracy index to measure the acuity during the comparison of magnitudes is the “Numeracy Screener” (Nosworthy et al., 2013). In this test, participants are required to compare pairs of both symbolic (56 digit pairs) and non-symbolic (pairs of dot arrays) magnitudes ranging from 1-9. The screener controls the visual characteristics of dot arrays by ensuring that half of them have equal area, while the other half have equal perimeter. Display time is controlled by giving participants a consistent time period (2 minutes) to complete the test (Nosworthy et al., 2013).

After demonstrating the validity of the Numeracy Screener by finding age-related improvements in the accuracy of numerical comparisons, the researchers (Nosworthy et al., 2013) tried to explore whether the test is capable of explaining variability in children’s arithmetic achievement. They found that the scores on both symbolic and non-symbolic items of this basic assessment tool significantly correlated with the scores on arithmetic and calculation skills, but only performance on the symbolic items accounts for the unique variance in arithmetic skills after controlling for the effect of some general cognitive abilities such as working memory, intelligence and reading ability.

### General Cognitive Factors and Their Relationship to Arithmetic Ability

In addition to numerical magnitude comparison processing as a domain-specific numerical ability, other domain-general cognitive factors might explain individual differences in children’s arithmetic abilities. Working memory, processing speed, and long-term memory are three cognitive abilities that may have a significant impact on individual differences in the arithmetic abilities of different children (Alloway & Alloway, 2010; Berg, 2008; Bull & Johnston, 1997; Dulaney, Vasilyeva, & O'Dwyer, 2015; Geary, 1993; Raghubar, Barnes, & Hecht, 2010). Before clarifying the plausible role of these cognitive factors in children’s arithmetic abilities, the development of arithmetic achievement in primary school–aged children should be briefly explained.

In the process of solving single-digit arithmetic problems, children pass through simpler to more advanced strategies. First, they learn to use a procedure in which they count all of the numbers, a procedure of obtaining a sum in which both addends are counted to find the solution. Then, they learn to use “counting on” (or min) procedures, in which they state the value of the larger addend before counting the smaller one. In both strategies, children may use their fingers for counting or count aloud. Finally, frequent use of counting strategies leads to the formation of long-term memory association between problems and their answers, eventually resulting in direct retrieval of answers from long-term memory when solving simple arithmetic problems (Geary, 1994; Siegler & Shrager, 1984). As mentioned earlier, numerical magnitude comparison skills may affect the ability of children to make decisions on the larger addend when using a min strategy (De Smedt et al., 2009). Furthermore, working memory, processing speed, and long-term memory are three types of cognitive ability that may play a central role as children progress through the different procedures used to solve arithmetic problems.

Working memory generally refers to a limited-capacity information-processing resource engaged in two key processes, the storage of information and the simultaneous processing of the same or other information (Berg, 2008). It is assumed that, in solving arithmetic problems, children use their working memory capacity to store relevant information (both addends) and simultaneously process that information using sum or min procedures. Furthermore, for the construction of an association between problem and answer in long-term memory, both addends (first and second number) and the final answer must be simultaneously active in working memory (Geary, 1993). Thus, children with higher working-memory capacity may solve simple arithmetic problems more efficiently than children with lower working-memory capacity. In concert with this assumption, several researchers examining the relationship between working memory and arithmetic ability have found that working memory uniquely explains individual differences in children’s arithmetic ability in the presence of other number-specific or general cognitive factors (e.g., De Smedt et al., 2009; Nosworthy et al., 2013; Rasmussen & Bisanz, 2005; Van der Ven, Van der Maas, Straatemeier, & Jansen, 2013).

Processing speed refers to the cognitive ability to execute simple cognitive tasks rapidly and efficiently (Case, 1985, as cited in Brown, 1986). During sum or min counting procedures to solve arithmetic problems, processing speed may facilitate counting speed to successfully pair problems and their answers in available working memory before decay. More efficient and faster pairing of problems and their answers in limited working-memory capacity results in more established long-term memory representations. In contrast, lower processing speed may increase the time necessary for deriving counted answers and for pairing a problem with its answer in working memory; hence, working memory may decay before the pairing is produced, impeding the development of an association in long-term memory (Geary, 1993). Bull and Johnston (1997) initially examined the role of processing speed in the arithmetic ability of children. They reported that processing speed uniquely contributed to arithmetic ability when other cognitive skills (including working memory and reading ability) also provided unique contributions. The unique contribution of processing speed to arithmetic ability has also been reported by other researchers (e.g., Durand, Hulme, Larkin, & Snowling, 2005; Fuchs et al., 2006; Kail & Hall, 1999; Swanson & Beebe-Frankenberger, 2004).

Given that arithmetic performance transparently depends on automatic retrieval of answers from long-term memory, long-term memory itself may be a plausible determinant of children’s arithmetic performance (Fuchs et al., 2006). However, given the role of working memory and processing speed in the retrieval of information from long-term memory, it is unknown to what degree arithmetic performance can be attributed to long-term memory rather than working memory and processing speed (Fuchs et al., 2006; Swanson & Beebe-Frankenberger, 2004).

### Present Study

In the present study, we examine the relationship between both the symbolic and the non-symbolic tasks of the Numeracy Screener and arithmetic ability in Iranian second grade children. Another goal is to find the extent to which these tasks explain the variability in both single-digit and two-digits arithmetic problem solving scores given the presence of contributions from working memory, processing speed, and long-term memory. With regard to the literature, we hypothesize that both symbolic and non-symbolic numerical magnitude comparisons are significantly correlated with arithmetic ability. Furthermore, we expect that symbolic (but not non-symbolic) magnitude comparisons uniquely explain the variability in arithmetic scores in the presence of working memory, processing speed, and long-term memory.

## Material and Methods

### Participants

For the initial sample, the subject group consisted of typically developing second-grade boys (*N* = 113) from four different classrooms. Using Raven’s Standard Progressive Test (Raven & Raven, 2003), the IQ level of the children ranged from 95 to 125. Every student in the sample was born in 2007. Their vision was normal or corrected to normal; none of them had developmental disabilities. After obtaining written informed parental consent, each subject was assessed individually during a 45-60 minute session in a quiet room located inside the school. All assessments were conducted in one session at the end of the academic year, when the students would have been taught all mathematical topics that were part of the curriculum for that year.

### Measures

#### Numerical Magnitude Comparisons

Numeracy Screener was used to measure both symbolic and non-symbolic numerical magnitude comparisons. The Numeracy Screener (Nosworthy et al., 2013) is a quick written tool to assess children’s ability to compare different numerical magnitudes ranging from 1-9. The magnitudes were presented in both symbolic (56 digit pairs) and non-symbolic (56 pairs of dot arrays) formats. In the non-symbolic form, dot stimuli were controlled for area and density; in both symbolic and non-symbolic formats, each numerical magnitude was counterbalanced taking into account the side of presentation. During the assessment section, participants were given one minute to cross out the larger of the two dot arrays in the non-symbol comparisons and one minute to cross out the larger of the two Arabic digits in the symbol comparisons. Before starting the assessment children were instructed to complete three sample items with the examiner and practice nine items on their own to ensure their understanding of the task. Furthermore, during the instruction given for the dot array comparisons, they were told not to count the dots, but rather to just try and estimate them. When the examiner ensured that the child understand the rule, the main part of the test was started. In addition, during the test, the child was reminded not to count the dots, but just try to estimate them as quickly as possible. The order in which children did the symbolic and non-symbolic representation comparisons was varied so that half of the students started with the Arabic digit comparison and the other half started with the dot array comparison. With administration of the Numeracy Screener, two scores were provided for each participant. The first score was the number of correct answers in the non-symbolic task (labeled as the non-symbolic comparison score); the second was the number of correct answers for the symbolic task (labeled as the symbolic comparison score).

#### Arithmetic Ability

Two written (paper and pencil) tasks were administered to measure each subject’s arithmetic ability. The two tests were a single-digit arithmetic problem solving test (labeled the “Math Fluency” test) and a two-digit arithmetic problem solving task (labeled as the “calculation” test). The math fluency test assessed the participant’s ability to quickly solve single-digit arithmetic problems. During the task, children were asked to solve 20 simple addition problems (5+4, 4+6, 9+3, 4+9, 3+7, 9+3, 8+5, 6+4, 9+5, 3+8, 4+5, 2+9, 8+6, 9+5, 7+8, 8+6, 4+5, 6+3, 9+6, 8+5) and 20 simple subtraction problems (6-4, 7-3, 9-7, 8-4, 9-6, 8-6, 9-3, 5-2, 8-3, 5-3, 9-5, 8-3, 7-4, 8-5, 9-2, 6-3, 8-4, 9-7, 7-5, 8-6); one minute was given for each section, during which children were to complete as many problems as possible. Half of the participants started with addition, half with subtraction. The number of correct answers for each task represented the score on that one.

The calculation task assessed the ability of participants to solve addition and subtraction problems involving two-digit numbers. Students were required to complete 10 problems in which they added two two-digit numbers (10+46, 28+32, 61+26, 59+15, 74+18, 12+15, 23+14, 16+32, 63+24, 81+18) and then to solve 10 similar subtraction problems (86-20, 34-14, 45-27, 60-45, 29-17, 90-15, 87-31, 56-23, 35-12, 43-18). The order in which the tasks were presented was similar to the order for the math fluency tasks.

Since two-digit arithmetic tasks may require more abstract reasoning, further attentional resources, and basic arithmetic skills, any time pressure may negatively affect child’s performance accuracy. Thus, unlike the math fluency problems, there was no time limitation in these tasks. The score on the tests was the number of correct answers for each task.

#### Working Memory

“Counting Span” is a well-known task to measure working-memory capacity. This task was originally presented by Case, Kurland, and Goldberg (1982) and was adapted by Keenan (1998). This task was used with some other modifications to adjust it to the ability of the participants. During the warm-up phase of the task, participants were told that they would be playing a memory game in which they had to count all of the red dots on a card and then remember the number of red dots. Each card had between two and six red dots and a number of distractor dots in blue and green. The task had six levels; each level had two trials. In the first level, the participant was presented one card at a time, told to count the red dots, and then asked how many red dots were on the card after the card was turned over. In the second level, the participant was shown one card and told to count its red dots. Subsequently, the card was turned over and another card was presented. The same procedure was repeated for the second card. Then, the participant was asked how many red dots were on the first card and the second card. In levels three through six, the procedure was exactly the same, but one more card was added to each level. The procedures terminated when the participant incorrectly answered twice at any level. The score was the number of correct answers across all levels, with a maximum of 12.

#### Processing Speed

The “WJ-III Visual Matching Task” (WJ is for “Woodcock-Johnson”) was used to measure processing speed (Woodcock, McGrew, Mather, & Schrank, 2003). In this task, the participants were required to locate and circle identical numbers in rows of six numbers. They had three minutes to complete 60 rows presented on two A4-sized pages. The participant’s score was the number of correctly circled matching numbers in each row. The test developer reported that the internal consistency of the task is .91.

#### Long-Term Memory

To measure semantic long-term memory, we used Retrieval Fluency, described in Woodcock and Johnson (1989). In this task, the participants were asked to recall related items divided between two categories (animals and fruits) for 1 minute per category. Credit was earned for each correct, non-duplicated answer.

## Results

The means and standard deviations (along with skewness and kurtosis) of all variables were presented in Table 1.

**Table 1:** Mean, Standard Deviation (SD), Range, Skewness, and Kurtosis of Variables

Variable	*N*	*M*	*SD*	Range	Skewness	Kurtosis
Symbolic Com	113	30.72	5.89	17-45	.0008	-.720
Non-Symbolic Com	113	30.21	5.83	14-41	-.7	.061
Two-Digit Addition	113	6.19	3.04	1-9	-.214	-.164
Two-Digit Subtraction	113	6.08	3.08	0-8	-.187	-.565
Single-Digit Addition	113	9.80	4.70	22-0	.445	-.164
Single-Digit Subtraction	113	7.50	3.40	0-18	.262	.903
Long-Term Memory	113	18.58	4.80	9-33	.418	.163
Working Memory	113	6.00	1.36	2-10	.476	.944
Processing Speed	113	8.74	2.55	2-15	-.154	-.061

*Note.* Com = Comparison.

As is shown in Table 1, all variables had skewness and kurtosis between -1 and +1. Bivariate correlations were conducted between the raw scores of all variables to determine their interrelationships. The results are presented in Table 2.

**Table 2:** Bivariate correlations between all variables

Variable	1	2	3	4	5	6	7	8	9
1. Symbolic Com	1	.61**	.18	.42**	.44**	.34**	.36**	.39**	.31*
2. Non-Symbolic Com		1	.17	.22*	.25*	.19*	.21*	.31*	.38**
3. Long-Term Memory			1	.22*	.17	.16	.14	.38**	.21*
4. Single-Digit Add				1	.60**	.38**	.58**	.43**	.46**
5. Single-Digit Sub					1	.56**	.49**	.34**	.32**
6. Two-Digit Add						1	.65**	.26*	.26*
7. Two-Digit Sub							1	.31*	.29*
8. Working Memory								1	.33**
9. Processing Speed									1

*Note.* Com = Comparison.

*p < .05. **p < .01.

Based on Cohen (1988), most variables had significant moderate to large correlations with each other (Table 2). With regard to the relationships between aptitude on numerical magnitude comparisons and arithmetic ability, the higher correlations were between symbolic magnitude comparisons and both single-digit addition and subtraction (.42 and. 44, respectively) and both two-digit addition and subtraction (.34 and .36, respectively). However, smaller correlations existed between non-symbolic magnitude comparison and all arithmetic tasks (.22, .25, .19, and .21). Regarding correlations between general cognitive abilities and arithmetic skills, single-digit addition and subtraction had significant correlations with long-term memory, working memory, and processing speed, while two-digit addition and subtraction were significantly correlated with working memory and processing speed, but not with long-term memory.

Since comparisons of both symbolic and non-symbolic magnitudes were moderately related to all arithmetic skills, in order to get more information about the unique contribution of each magnitude comparison to both math fluency and calculation skills, four linear regression analyses were conducted, two for single-digit addition and subtraction and two for two-digit addition and subtraction. The major focus of these analyses was to carefully examine the independence of each numerical magnitude comparison to predict single-digit and two digits addition and subtraction skills while controlling for the effect of working memory, processing speed, and long-term memory. For this purpose, all of the variables were entered in one step. The results were presented in Table 3 and Table 4.

**Table 3:** Linear Regression Analyses With Single-Digit Addition and Subtraction as Dependent Variables

	Single-Digit Addition *F* = 9.563, *p* < .001, *R*^2^ = .31		Single-Digit Subtraction *F* = 6.110, *p* < .001, *R^2^* = .23	
Predictor	ß	*t*	ß	*t*
Working Memory	.175*	2.02	.272*	2.90
Processing Speed	.279*	2.89	.250*	2.46
Long-Term Memory	.066	0.74	.006	-0.04
Non-Symbolic Com	-.095	-0.89	.015	0.09
Symbolic Com	.320*	3.03	.212*	1.89

*Note.* Com = Comparison.

*p < .05.

**Table 4:** Linear Regression Analyses With Two-Digit Addition and Subtraction as Dependent Variables

	Two-Digit Addition *F* = 5.322, *p* < .001, *R^2^* = .22		Two-Digit Subtraction *F* = 6.321, *p* < .001, *R^2^* = .24	
Predictor	ß	*t*	ß	*t*
Working Memory	.210*	2.45	.197*	1.69
Processing Speed	.043	0.43	-.054	0.51
Long-Term Memory	.039	0.50	.047	0.54
Non-Symbolic Com	-.050	-0.05	-.049	-0.47
Symbolic Com	.501*	3.01	.444*	4.21

*Note.* Com = Comparison.

*p < .05.

Table 3 shows that the first linear regression analysis with single-digit addition as the dependent variable was significant (*F* (5, 113) = 9.563, *p* < .001, *R^2^* = .31). In this model, performance on working memory, processing speed, and symbolic comparison explained the significant and unique variance in single digit addition. Conversely, non-symbolic magnitude comparison and long-term memory did not uniquely explain the variance in that. The second linear regression analysis, which used single-digit subtraction as a dependent variable, was also significant (*F* (5, 113) = 6.211, *p* < .001, *R*^2^ = .23). In this model, similar to first model, symbolic comparison, working memory, and processing speed had significant and unique contributions to single-digit subtraction. However, non-symbolic comparison and long-term memory did not account for significant and unique contributions to (or variances in) single-digit subtraction skill.

Table 4 shows that the third linear regression analysis with two-digit addition as the dependent variable was significant (*F* (5, 113) = 5.322, *p* < .001, *R^2^* = .22). In this model, performance on working memory, and symbolic comparison explained the significant and unique variance in two- digit addition. Conversely, non-symbolic magnitude comparison, processing speed, and long-term memory did not uniquely explain the variance in that. The forth linear regression analysis, which used two-digit subtraction as a dependent variable, was also significant (*F* (5, 113) = 6.321, *p* < .001, *R^2^* = .24). In this model, similar to third model, symbolic comparison and working memory had significant and unique contributions two-digit subtraction. However, non-symbolic comparison, processing speed, and long-term memory did not account for significant and unique contributions to (or variances in) two-digit subtraction skill.

## Discussion and Conclusion

The present study explored whether performance on the symbolic and non-symbolic items of a Numeracy Screener is related to individual differences in the arithmetic ability of second grade children in Iran. The arithmetic skills measured included single-digit addition and subtraction tasks and series of two-digit addition and subtraction problems. Furthermore, we intended to determine whether magnitude comparison tasks significantly explain variances of single-digit and two-digit addition and subtraction skills over some general cognitive factors including working memory, processing speed, and long-term memory.

We found that participants’ scores on both symbolic and non-symbolic items were significantly correlated with scores on single-digit and two-digit arithmetic. Participants who scored highly on symbolic or non-symbolic magnitude comparison tasks on the Numeracy Screener also tended to receive high scores on single-digit and two-digit arithmetic. Nevertheless, the results of our regression analyses demonstrated that only participants’ scores on the symbolic items of the Numeracy Screener uniquely explain the variances in both single-digit and two-digit addition and subtraction skills. These findings are closely comparable with the results reported by Nosworthy and colleagues (2013). These researchers initially used the Numeracy Screener to measure performance on numerical magnitude comparisons and determine its relation to arithmetic ability. In addition, similar results have also been reported by researchers from studies that used other forms of magnitude comparison tasks (e.g., Holloway & Ansari, 2009; Kolkman et al., 2013; Lonnemann et al., 2011; Mundy & Gilmore, 2009; Sasanguie et al., 2012; Vanbinst et al., 2015). For example, in a recent study Vanbinst et al. (2015) tried to explore the relationship between numerical magnitude comparison ability and simple arithmetic ability in first- and second-grade children using a computerized version of the magnitude comparison task. The researchers (Vanbinst et al., 2015) used accuracy and reaction time as indices to measure both symbolic and non-symbolic magnitude comparison. Their results showed that symbolic (but not non-symbolic) numerical magnitude comparisons uniquely explain the variances in arithmetic ability among primary school children in the first grade.

Our data also support the commonly held assumption that symbolic magnitudes are acquired through a process in which the mapping symbolic system is mapped onto a previously existing non-symbolic system (Mundy & Gilmore, 2009). The significant correlation between our symbolic and non-symbolic comparison tasks, as well as the significant relationships between both magnitude comparisons and arithmetic ability, suggests that similar developmental trajectories may underlie the processing and representation of both symbolic and non-symbolic numerical magnitudes. However, the unique contribution of symbolic magnitude comparison to arithmetic ability may suggest that symbolic magnitude comparisons cover the variances explained by non-symbolic representations.

The results of our regression analyses also revealed that working memory uniquely explains the variance in both single-digit and two-digit arithmetic, in the presence of the unique contribution of symbolic magnitude comparison. The central role of working memory in developing children’s arithmetic ability have been reported by several studies (e.g., Dulaney et al., 2015; Raghubar et al., 2010; Van der Ven et al., 2013). It has been assumed that working-memory capacity provides a limited work space for children to use sum or min strategies when solving simple digit arithmetic problems, consequently leading to the development of associations between problems and their answers in long-term memory (Geary, 1993). Thus, higher working-memory capacity leads to more accurate performance while making calculations and more long-term memory representations. In addition, attentional resource of working memory can assist children as they attempt to perform accurate two-digit calculations. In concert with this assumption and in line with previous studies, our findings support the vital role of working-memory capacity in children’s arithmetic achievement.

To elaborate further, the regression analyses revealed that processing speed uniquely contributed to single-digit and two-digit addition and subtraction, but not to two-digit arithmetic. It has been assumed that, in solving arithmetic problems, processing speed may facilitate counting speed to successfully pair problems and their answers in available working memory capacity before decay begins. More efficient and faster pairing of problems and their answers in limited working memory capacity result in more established long-term memory representations (Geary, 1993). Bull and Johnston (1997) initially examined the role of processing speed in children’s simple digit arithmetic abilities. They reported that processing speed uniquely contributed to arithmetic ability in the presence of unique contributions of other cognitive skills. Furthermore, the unique contribution of processing speed to simple-digit arithmetic abilities has also been reported by other researchers (e.g., Durand et al., 2005; Fuchs et al., 2006; Kail & Hall, 1999; Swanson & Beebe-Frankenberger, 2004). In line with Bull and Johnston (1997), the present study also supports the unique contribution of processing speed to simple-digit arithmetic problem solving. In addition, both the single-digit arithmetic test and the processing speed task (visual matching) were paper-and-pencil, time-restricted tasks. Thus, the rate of children’s sensory-motor performance may underpin their execution speed in both tasks. However, unlike single-digit arithmetic, participants had an unlimited amount of time to complete our two-digit arithmetic test. This may clarify the reason for the insignificant contribution of processing speed to calculation. In addition, compared with simple (single-digit) arithmetic, solving problems involving two-digit arithmetic may require more abstract reasoning, further attentional resources, and basic arithmetic skills. Thus, it might be expected that the variances in two-digit arithmetic are explained to a greater degree by fluid intelligence, working memory, or basic arithmetic abilities rather than processing speed.

In line with Swanson and Beebe-Frankenberger (2004) and Fuchs et al. (2006), our regression analyses revealed that long-term memory did not uniquely contribute to variances in single-digit or two-digit arithmetic. These findings are in contrast with the assumption that long-term memory itself may be a plausible determinant of arithmetic performance in children. Given the role of working memory in the retrieval of information from long-term memory, it is plausible that arithmetic performance can be attributed to working memory rather than semantic long-term memory.

In conclusion, the current findings demonstrated that significant relationships exist between the performances of second-grade primary school children in Iran on the Numeracy Screener items as well as on both measured arithmetic abilities (single-digit and two-digit arithmetic). In addition, it was found that only the symbolic comparison portion of the Numeracy Screener accounted for unique variances in both single-digit and two-digit arithmetic. These results are in line with previous research in which the Numeracy Screener was initially used (Nosworthy et al., 2013) and highlights the applicability of the test in educational setting. Quickly and easily assessing the numerical magnitude processing abilities of students using such a simple and easily available written test can help educators focus on these essential skills during math instruction in the classroom. In particular, this may assist educators in developing countries where there is no available advanced computerized equipment in many school settings. Furthermore, the significant relationship shown between non-symbolic items of the Numeracy Screener and both single-digit and two-digit arithmetic skill replicates the findings of Nosworthy and colleagues (2013) and may suggest that the non-symbolic portion of the test could be utilized by itself for preschool children who do not have semantic representations of number symbols. The performance of preschool children on the non-symbolic items of the Numeracy Screener may explain their individual differences in mathematic ability during the primary school years. Further studies are recommended to investigate this line of research.

The current findings demonstrated that a combination of numerical magnitude comparison tasks assessed via a Numeracy Screener, memory, and processing speed explain 30% and 22% of the variances in math fluency and calculation skill, respectively. The remaining variances might be explained by other domain-specific factors such as number line estimation, subitizing ability, counting knowledge, or other domain-general cognitive abilities such as attentive behavior, inhibition, task switching, language, and intelligence. Further research is recommended to determine the relationship between the Numeracy Screener, the other factors summed up above, and arithmetic ability in primary school children, especially in other developing countries.

## References

[r1] Alloway T. P. Alloway R. G. (2010). Investigating the predictive roles of working memory and IQ in academic attainment. Journal of Experimental Child Psychology, 106(1), 20–29. 10.1016/j.jecp.2009.11.003 20018296

[r2] Barnea-Goraly N. Eliez S. Menon V. Bammer R. Reiss A. L. (2005). Arithmetic ability and parietal alterations: A diffusion tensor imaging study in velocardiofacial syndrome. Cognitive Brain Research, 25(3), 735–740. 10.1016/j.cogbrainres.2005.09.013 16260124

[r3] Barth H. Kanwisher N. Spelke E. (2003). The construction of large number representations in adults. Cognition, 86(3), 201–221. 10.1016/S0010-0277(02)00178-6 12485738

[r4] Berg D. H. (2008). Working memory and arithmetic calculation in children: The contributory roles of processing speed, short-term memory, and reading. Journal of Experimental Child Psychology, 99(4), 288–308. 10.1016/j.jecp.2007.12.002 18242631

[r5] Bonny J. W. Lourenco S. F. (2013). The approximate number system and its relation to early math achievement: Evidence from the preschool years. Journal of Experimental Child Psychology, 114(3), 375–388. 10.1016/j.jecp.2012.09.015 23201156 PMC3570698

[r6] Brankaer C. Ghesquière P. De Smedt B. (2014). Numerical magnitude processing deficits in children with mathematical difficulties are independent of intelligence. Research in Developmental Disabilities, 35(11), 2603–2613. 10.1016/j.ridd.2014.06.022 25036314

[r7] Brown G. (1986). Case, R. (1985). Intellectual development: Birth to adulthood [Essay review]. The British Journal of Educational Psychology, 56, 220–222. doi:. 10.1111/j.2044-8279.1986.tb02666.x

[r8] Bugden S. Price G. R. McLean D. A. Ansari D. (2012). The role of the left intraparietal sulcus in the relationship between symbolic number processing and children's arithmetic competence. Developmental Cognitive Neuroscience, 2(4), 448–457. 10.1016/j.dcn.2012.04.001 22591861 PMC7005765

[r9] Bull R. Johnston R. S. (1997). Children's arithmetical difficulties: Contributions from processing speed, item identification, and short-term memory. Journal of Experimental Child Psychology, 65(1), 1–24. 10.1006/jecp.1996.2358 9126630

[r10] Case R. Kurland D. M. Goldberg J. (1982). Operational efficiency and the growth of short-term memory span. Journal of Experimental Child Psychology, 33(3), 386–404. 10.1016/0022-0965(82)90054-6

[r11] Caviola S. Mammarella I. C. Lucangeli D. Cornoldi C. (2014). Working memory and domain-specific precursors predicting success in learning written subtraction problems. Learning and Individual Differences, 36, 92–100. 10.1016/j.lindif.2014.10.010

[r12] Chen Q. Li J. (2014). Association between individual differences in non-symbolic number acuity and math performance: A meta-analysis. Acta Psychologica, 148, 163–172. 10.1016/j.actpsy.2014.01.016 24583622

[r13] Cohen, J. (1988). *Statistical power analysis for the behavioral sciences* (2nd ed.). Hillsdale, NJ, USA: Lawrence Erlbaum Associates.

[r14] Defever E. De Smedt B. Reynvoet B. (2013). Numerical matching judgments in children with mathematical learning disabilities. Research in Developmental Disabilities, 34(10), 3182–3189. 10.1016/j.ridd.2013.06.018 23886760

[r15] Deheane, S. (2011). *The number sense: How the mind creates mathematics* (Revised and expanded ed.). New York, NY, USA: Oxford University Press.

[r16] De Smedt B. Holloway I. D. Ansari D. (2011). Effects of problem size and arithmetic operation on brain activation during calculation in children with varying levels of arithmetical fluency. NeuroImage, 57(3), 771–781. 10.1016/j.neuroimage.2010.12.037 21182966

[r17] De Smedt B. Noël M.-P. Gilmore C. Ansari D. (2013). How do symbolic and non-symbolic numerical magnitude processing skills relate to individual differences in children's mathematical skills? A review of evidence from brain and behavior. Trends in Neuroscience and Education, 2(2), 48–55. 10.1016/j.tine.2013.06.001

[r18] De Smedt B. Verschaffel L. Ghesquière P. (2009). The predictive value of numerical magnitude comparison for individual differences in mathematics achievement. Journal of Experimental Child Psychology, 103(4), 469–479. 10.1016/j.jecp.2009.01.010 19285682

[r19] Dulaney A. Vasilyeva M. O'Dwyer L. (2015). Individual differences in cognitive resources and elementary school mathematics achievement: Examining the roles of storage and attention. Learning and Individual Differences, 37, 55–63. 10.1016/j.lindif.2014.11.008

[r20] Durand M. Hulme C. Larkin R. Snowling M. (2005). The cognitive foundations of reading and arithmetic skills in 7-to 10-year-olds. Journal of Experimental Child Psychology, 91(2), 113–136. 10.1016/j.jecp.2005.01.003 15890173

[r21] Fuchs L. S. Fuchs D. Compton D. L. Powell S. R. Seethaler P. M. Capizzi A. M. Fletcher J. M. (2006). The cognitive correlates of third-grade skill in arithmetic, algorithmic computation, and arithmetic word problems. Journal of Educational Psychology, 98(1), 29–43. 10.1037/0022-0663.98.1.29

[r22] Fuchs L. S. Geary D. C. Compton D. L. Fuchs D. Hamlett C. L. Bryant J. D. (2010). The contributions of numerosity and domain-general abilities to school readiness. Child Development, 81(5), 1520–1533. 10.1111/j.1467-8624.2010.01489.x 20840238 PMC2941220

[r23] Fuhs M. W. McNeil N. M. (2013). ANS acuity and mathematics ability in preschoolers from low-income homes: Contributions of inhibitory control. Developmental Science, 16(1), 136–148. 10.1111/desc.12013 23278935

[r24] Geary D. C. (1993). Mathematical disabilities: Cognitive, neuropsychological, and genetic components. Psychological Bulletin, 114(2), 345–362. 10.1037/0033-2909.114.2.345 8416036

[r25] Geary, D. C. (1994). *Children's mathematical development: Research and practical applications*: American Psychological Association.

[r26] Geary D. C. Hoard M. K. Nugent L. Byrd-Craven J. (2008). Development of number line representations in children with mathematical learning disability. Developmental Neuropsychology, 33(3), 277–299. 10.1080/87565640801982361 18473200 PMC4439204

[r27] Gilmore C. Attridge N. Inglis M. (2011). Measuring the approximate number system. Quarterly Journal of Experimental Psychology, 64(11), 2099–2109. 10.1080/17470218.2011.574710 21846265

[r28] Halberda J. Feigenson L. (2008). Developmental change in the acuity of the “Number Sense”: The Approximate Number System in 3-, 4-, 5-, and 6-year-olds and adults. Developmental Psychology, 44(5), 1457–1465. 10.1037/a0012682 18793076

[r29] Halberda J. Mazzocco M. M. Feigenson L. (2008). Individual differences in non-verbal number acuity correlate with maths achievement. Nature, 455(7213), 665–668. 10.1038/nature07246 18776888

[r30] Heine A. Wißmann J. Tamm S. De Smedt B. Schneider M. Stern E. Jacobs A. M. (2013). An electrophysiological investigation of non-symbolic magnitude processing: Numerical distance effects in children with and without mathematical learning disabilities. Cortex*,* 49(8), 2162-2177. 10.1016/j.cortex.2012.11.009 23287447

[r31] Holloway I. D. Ansari D. (2009). Mapping numerical magnitudes onto symbols: The numerical distance effect and individual differences in children’s mathematics achievement. Journal of Experimental Child Psychology, 103(1), 17–29. 10.1016/j.jecp.2008.04.001 18513738

[r32] Holloway I. D. Ansari D. (2010). Developmental specialization in the right intraparietal sulcus for the abstract representation of numerical magnitude. Journal of Cognitive Neuroscience, 22(11), 2627–2637. 10.1162/jocn.2009.21399 19929327

[r33] Inglis M. Attridge N. Batchelor S. Gilmore C. (2011). Non-verbal number acuity correlates with symbolic mathematics achievement: But only in children. Psychonomic Bulletin & Review, 18(6), 1222–1229. 10.3758/s13423-011-0154-1 21898191

[r34] Inglis M. Gilmore C. (2014). Indexing the approximate number system. Acta Psychologica, 145, 147–155. 10.1016/j.actpsy.2013.11.009 24361686

[r35] Isaacs E. B. Edmonds C. J. Lucas A. Gadian D. G. (2001). Calculation difficulties in children of very low birthweight: A neural correlate. Brain, 124(9), 1701–1707. 10.1093/brain/124.9.1701 11522573

[r36] Kail R. Hall L. K. (1999). Sources of developmental change in children's word-problem performance. Journal of Educational Psychology, 91(4), 660–668. 10.1037/0022-0663.91.4.660

[r37] Keenan T. (1998). Memory span as a predictor of false belief understanding. New Zealand Journal of Psychology, 27, 36–43.

[r38] Kolkman M. E. Kroesbergen E. H. Leseman P. P. (2013). Early numerical development and the role of non-symbolic and symbolic skills. Learning and Instruction, 25, 95–103. 10.1016/j.learninstruc.2012.12.001

[r39] Krajewski K. Schneider W. (2009). Exploring the impact of phonological awareness, visual–spatial working memory, and preschool quantity–number competencies on mathematics achievement in elementary school: Findings from a 3-year longitudinal study. Journal of Experimental Child Psychology, 103(4), 516–531. 10.1016/j.jecp.2009.03.009 19427646

[r40] Landerl K. Bevan A. Butterworth B. (2004). Developmental dyscalculia and basic numerical capacities: A study of 8–9-year-old students. Cognition, 93(2), 99–125. 10.1016/j.cognition.2003.11.004 15147931

[r41] Lindskog M. Winman A. Juslin P. Poom L. (2013). Measuring acuity of the approximate number system reliably and validly: The evaluation of an adaptive test procedure. Frontiers in Psychology, 4, 510. 10.3389/fpsyg.2013.00510 23964256 PMC3734355

[r42] Linsen S. Verschaffel L. Reynvoet B. De Smedt B. (2014). The association between children's numerical magnitude processing and mental multi-digit subtraction. Acta Psychologica, 145, 75–83. 10.1016/j.actpsy.2013.10.008 24296255

[r43] Lipton J. S. Spelke E. S. (2003). Origins of number sense large-number discrimination in human infants. Psychological Science, 14(5), 396–401. 10.1111/1467-9280.01453 12930467

[r44] Lonnemann J. Linkersdörfer J. Hasselhorn M. Lindberg S. (2011). Symbolic and non-symbolic distance effects in children and their connection with arithmetic skills. Journal of Neurolinguistics, 24(5), 583–591. 10.1016/j.jneuroling.2011.02.004

[r45] Lourenco S. F. Bonny J. W. Fernandez E. P. Rao S. (2012). Nonsymbolic number and cumulative area representations contribute shared and unique variance to symbolic math competence. Proceedings of the National Academy of Sciences of the United States of America, 109(46), 18737–18742. 10.1073/pnas.1207212109 23091023 PMC3503215

[r46] Lyons I. M. Ansari D. Beilock S. L. (2015). Qualitatively different coding of symbolic and nonsymbolic numbers in the human brain. Human Brain Mapping, 36(2), 475–488. 10.1002/hbm.22641 25238646 PMC6869776

[r47] Maloney E. A. Risko E. F. Preston F. Ansari D. Fugelsang J. (2010). Challenging the reliability and validity of cognitive measures: The case of the numerical distance effect. Acta Psychologica, 134(2), 154–161. 10.1016/j.actpsy.2010.01.006 20185118

[r48] Mazzocco M. M. Feigenson L. Halberda J. (2011). Preschoolers’ precision of the approximate number system predicts later school mathematics performance. PLOS ONE, 6(9), e23749. 10.1371/journal.pone.0023749 21935362 PMC3173357

[r49] Merkley R. Ansari D. (2010). Using eye tracking to study numerical cognition: The case of the ratio effect. Experimental Brain Research, 206(4), 455–460. 10.1007/s00221-010-2419-8 20862461

[r50] Mundy E. Gilmore C. K. (2009). Children’s mapping between symbolic and nonsymbolic representations of number. Journal of Experimental Child Psychology, 103(4), 490–502. 10.1016/j.jecp.2009.02.003 19327782

[r51] Nosworthy N. Bugden S. Archibald L. Evans B. Ansari D. (2013). A two-minute paper-and-pencil test of symbolic and nonsymbolic numerical magnitude processing explains variability in primary school children's arithmetic competence. PLOS ONE, 8(7), e67918. 10.1371/journal.pone.0067918 23844126 PMC3699460

[r52] Östergren R. Träff U. (2013). Early number knowledge and cognitive ability affect early arithmetic ability. Journal of Experimental Child Psychology, 115(3), 405–421. 10.1016/j.jecp.2013.03.007 23665177

[r53] Passolunghi M. C. Lanfranchi S. (2012). Domain-specific and domain-general precursors of mathematical achievement: A longitudinal study from kindergarten to first grade. The British Journal of Educational Psychology, 82(1), 42–63. 10.1111/j.2044-8279.2011.02039.x 22429058

[r54] Price G. R. Holloway I. Räsänen P. Vesterinen M. Ansari D. (2007). Impaired parietal magnitude processing in developmental dyscalculia. Current Biology, 17(24), R1042–R1043. 10.1016/j.cub.2007.10.013 18088583

[r55] Price G. R. Mazzocco M. M. Ansari D. (2013). Why mental arithmetic counts: Brain activation during single digit arithmetic predicts high school math scores. The Journal of Neuroscience, 33(1), 156–163. 10.1523/JNEUROSCI.2936-12.2013 23283330 PMC6618631

[r56] Price G. R. Palmer D. Battista C. Ansari D. (2012). Nonsymbolic numerical magnitude comparison: Reliability and validity of different task variants and outcome measures, and their relationship to arithmetic achievement in adults. Acta Psychologica, 140(1), 50–57. 10.1016/j.actpsy.2012.02.008 22445770

[r57] Raghubar K. P. Barnes M. A. Hecht S. A. (2010). Working memory and mathematics: A review of developmental, individual difference, and cognitive approaches. Learning and Individual Differences, 20(2), 110–122. 10.1016/j.lindif.2009.10.005

[r58] Rasmussen C. Bisanz J. (2005). Representation and working memory in early arithmetic. Journal of Experimental Child Psychology, 91(2), 137–157. 10.1016/j.jecp.2005.01.004 15890174

[r59] Raven, J., & Raven, J. (2003). Raven progressive matrices. In R. S. McCallum (Ed.), *Handbook of nonverbal assessment* (pp. 223-237). New York, NY, USA: Springer.

[r60] Rousselle L. Noël M.-P. (2007). Basic numerical skills in children with mathematics learning disabilities: A comparison of symbolic vs non-symbolic number magnitude processing. Cognition, 102(3), 361–395. 10.1016/j.cognition.2006.01.005 16488405

[r61] Sasanguie D. Göbel S. M. Moll K. Smets K. Reynvoet B. (2013). Approximate number sense, symbolic number processing, or number–space mappings: What underlies mathematics achievement? Journal of Experimental Child Psychology, 114(3), 418–431. 10.1016/j.jecp.2012.10.012 23270796

[r62] Sasanguie D. Van den Bussche E. Reynvoet B. (2012). Predictors for mathematics achievement? Evidence from a longitudinal study. Mind, Brain, and Education, 6(3), 119–128. 10.1111/j.1751-228X.2012.01147.x

[r63] Siegler, R. S., & Shrager, J. (1984). Strategy choices in addition and subtraction: How do children know what to do? *Origins of cognitive skills: The 18th Annual Carnegie Mellon Symposium on Cognition* (pp. 229-293). Hillsdale, NJ: USA: Erlbaum.

[r64] Soltész F. Szűcs D. Szűcs L. (2010). Relationships between magnitude representation, counting and memory in 4- to 7-year-old children: A developmental study. Behavioral and Brain Functions, 6, 13. 10.1186/1744-9081-6-13 20167066 PMC2833140

[r65] Swanson H. L. Beebe-Frankenberger M. (2004). The relationship between working memory and mathematical problem solving in children at risk and not at risk for serious math difficulties. Journal of Educational Psychology, 96(3), 471–491. 10.1037/0022-0663.96.3.471

[r66] Träff U. (2013). The contribution of general cognitive abilities and number abilities to different aspects of mathematics in children. Journal of Experimental Child Psychology, 116(2), 139–156. 10.1016/j.jecp.2013.04.007 23773916

[r67] Vanbinst K. Ansari D. Ghesquière P. De Smedt B. (2016). Symbolic numerical magnitude processing is as important to arithmetic as phonological awareness is to reading. PLoS ONE, 11(3), e0151045. 10.1371/journal.pone.0151045 26942935 PMC4778857

[r68] Vanbinst K. Ghesquière P. De Smedt B. (2012). Numerical magnitude representations and individual differences in children's arithmetic strategy use. Mind, Brain, and Education, 6(3), 129–136. 10.1111/j.1751-228X.2012.01148.x

[r69] Vanbinst K. Ghesquière P. De Smedt B. (2015). Does numerical processing uniquely predict first graders’ future development of single-digit arithmetic? Learning and Individual Differences, 37, 153–160. 10.1016/j.lindif.2014.12.004

[r70] Van der Ven S. H. G. Van der Maas H. L. J. Straatemeier M. Jansen B. R. J. (2013). Visuospatial working memory and mathematical ability at different ages throughout primary school. Learning and Individual Differences, 27, 182–192. 10.1016/j.lindif.2013.09.003

[r71] Woodcock, R. W., & Johnson, M. B. (1989). *WJ-R Tests of Cognitive Ability.* Itasca, IL, USA: Riverside Publishing.

[r72] Woodcock, R. W., McGrew, K. S., Mather, N., & Schrank, F. A. (2003). *Woodcock-Johnson III diagnostic supplement to the tests of cognitive abilities.* Itasca, IL, USA: Riverside Publishing.

[r73] Xu, F., & Spelke, E. S. (2000). Large number discrimination in 6-month-old infants. *Cognition, 74*(1), B1-B11. 10.1016/s0010-0277(99)00066-9 10594312

